# Investigation the Possibility of Using Peptides with a Helical Repeating Pattern of Hydro-Phobic and Hydrophilic Residues to Inhibit IL-10

**DOI:** 10.1371/journal.pone.0153939

**Published:** 2016-04-21

**Authors:** Guoying Ni, Shu Chen, Yuedong Yang, Scott F. Cummins, Jian Zhan, Zhixiu Li, Bin Zhu, Kate Mounsey, Shelley Walton, Ming Q. Wei, Yuejian Wang, Yaoqi Zhou, Tianfang Wang, Xiaosong Liu

**Affiliations:** 1 Genecology Research Centre, University of the Sunshine Coast, Maroochydore DC, Australia; 2 Inflammation and Healing Research Cluster, School of Health and Sport Sciences, University of Sunshine Coast, Maroochydore DC, Australia; 3 School of Medical Science, Griffith Health Institute, Griffith University, Gold Coast, Australia; 4 The Institute for Glycomics and School of Information and Communication Technology, Griffith University, Gold Coast, Australia; 5 Diamantina Institute, University of Queensland, Woolloongabba, Australia; 6 Cancer Research Institute, Foshan First People’s Hospital, Foshan, Guangdong, China; Second University of Naples, ITALY

## Abstract

Blockade of IL-10 signalling clears chronic viral and bacterial infections. Immunization together with blockade of IL-10 signalling or relatively low level of IL-10 further enhances viral and bacterial clearance. IL-10 functions through binding to interleukin 10 receptor (IL-10R). Here we showed that peptides P1 and P2 with the hydrophobic and hydrophilic pattern of the IL10R-binding helix in IL-10 could bind with either IL-10R1 or IL-10, and inhibit inflammatory signals with long duration and negligible cytotoxicity *in vitro*. Furthermore, P2 can enhance antigen specific CD8+ T cell responses in mice induced by the vaccine based on a long peptide of protein E7 in a human papillomavirus type 16.

## Introduction

Interleukin 10 (IL-10) is an anti-inflammatory cytokine with multiple biological functions. Its expression level is elevated during persistent viral infections such as human immunodeficiency virus (HIV), hepatitis B and hepatitis C (HBV, HCV) in humans [1–3]. IL-10 blockade has been shown to enhance T cell responses which in turn control persistent infection of lymphocytic choriomeningitis virus or cytomegalovirus [3–5] in mice. Immunization in conjunction with blockade of IL-10 signalling increases vaccine-induced T cell responses and clears bacteria, parasite and chronic viral infection more efficiently than that with IL-10/IL-10R at normal or over-expression levels[2, 6–9]. In addition, blocking IL-10 signalling at the time of immunization is able to control tumour growth in mouse model [10, 11]. IL-10 knockout mice develops chronic inflammation in intestine only in late life stage, suggesting that temporal blocking of IL-10 signalling or lowering IL-10 level may not cause severe side effects if used in clinic. Therefore, the inhibition of IL-10 may become an exciting new strategy to control chronic viral infection and related cancers.

IL-10 signals through IL-10 receptor (IL-10R). IL-10R is a class II cytokine family member composed of IL-10R1 and IL-10R2 subunits. IL-10R1 binds to IL-10 with high affinity while IL-10R2 is an accessory subunit for signal transduction [12–14]. *In vivo* inhibition of IL-10 signalling can be achieved through administration of anti-IL-10R antibodies [15, 16]. However, clinical grade humanized antibodies are not available for clinical use [17].

Compared to small molecules, biologic drugs such as peptides, nucleotides and proteins possess the advantages of target-sensitivity, low cytotoxicity, and eco-friendliness [18, 19]. Some attempts have been made to develop large biomolecules into IL10 inhibitors [20, 21]. For example, the extracellular domain of human IL-10R1/Fc regions of human IgG1 heavy chain is able to inhibit IL-10 function *in vitro*. Inhibitory peptides of IL-10 signalling *in vitro* and *in vivo* have also been discovered by phage display techniques. An oligonucleotide aptamer uncovered by using high throughput sequencing can inhibit a murine CT 26 tumour growth similar to the effects mediated by anti-IL-10R antibodies [11]. However, above experimental techniques for peptide discovery are costly and labour intensive. As a result, computational approaches have been developed to prioritise candidates for experimental validations.

There are two main computational approaches based on ligand activity and receptor structures, respectively [22–25]. Ligand-based approaches infer new ligands based on the quantitative relation between inhibition activity and physio-chemical and structural information of known ligands. Structure-based approach, on the other hand, relies on the 3-dimensional structures (X-ray crystallography or NMR spectroscopy) of biological targets to search for the best inhibitors based on the highest binding interactions between the candidate inhibitors and the target.

Structure-based drug design has been becoming increasingly successful. For example, a group of βpep peptides (antiparallel β-sheet structure and a preponderance of positively charged and hydrophobic residues) were designed and screened for the ability to inhibit endothelial cell (EC) proliferation, an *in vitro* indicator of angiogenic potential; out of 30 designed peptides, one potent angiogenesis inhibitor was found more effective than other well-known antiangiogenics [26]. A gluten peptide analogue was designed successfully as a tight-binding ligand for HLA-DQ2 (one of the two pharmacological targets of celiac sprue) [27]. The structure of circumsporozoite protein (CSP) of *Plasmodium falciparum*, a leading candidate antigen for inclusion in a malaria subunit vaccine, was employed to design a peptide UK-39. The intramuscular delivery of UK-39 to mice and rabbits elicits IgG antibodies, which in turn inhibited invasion of hepatocytes by *P*. *falciparum sporozoites*. This is an example of the rational development of a malaria vaccine [28]. A peptide was designed to inhibit urokinase-type plasminogen activator receptor (uPAR), which plays critical role in cancer cell growth, survival, invasion, and metastasis can inhibit cell migration and lung metastasis [29]. A group of designed peptides containing an aldehyde at the C terminus can inhibit syndrome (SARS) chymotrypsin-like protease (3CL) with an IC50 value of 98 nM [30]. Several designed peptide ligands were also found to enhance MHC binding and hence T cell recognition of gp100 in HLA-DR4^+^ melanoma patients [31].

Most of studies designed drug candidates (peptides or small molecules) directly using the crystal or NMR structures of the targets proteins or receptor/ligand binding areas, also in concert with molecular dynamics simulation in some cases, to elucidate the key structural characteristics, such as α-turn [29], β-sheet [26, 28], hydrogen bonding between side chain of the ligand and receptor [27], the conserved peptide region of the target [32], steric space of the binding area of the target [30], hydrophobicity of the residues within the binding area [31], and so forth; then, molecular moieties (e.g., amino acids, nucleic acids and chemical groups etc.) can be chosen with emphasis on one or multiple characteristics, to build up the drug molecules, which would be validated by further bioassays.

In the current paper, we designed two peptides (P1 and P2) that can inhibit IL-10 via structure-based analysis, focusing on the helix structure of the ligand protein (IL-10) within the binding area of IL-10/IL-10R, as well as the amino acid pattern of the helix sequence. More specifically, we obtained our peptides based on physio-chemical properties of the IL10 peptide segments in the interface of the IL-10/IL-10R complex structure. *In vitro* tests confirmed the usefulness of the designed peptides in inhibiting IL-10 level; more significantly, the *ex vivo* assay also suggested that one designed peptide could enhance the CD8+ T cell responses using a mouse model.

## Materials and Methods

### Mice

We purchased 6–8 weeks old adult female C57BL/6 (H-2^b^) mice that are specific pathogen free (SPF) from the Animal Resource Centre, Sun Yat-Sen University, Guangdong province, China and kept them under SPF conditions with irradiated food and autoclaved water, and with cycles of light and dark of 12 hours at the centre. Mice were randomly separated into groups of 3–5 mice in each cage. No animals became sick or died prior to the experimental endpoint. The mice were euthanized with cervical dislocation according to the hospital’s AEC protocol. All experiments were approved by and performed in compliance with the guidelines of Foshan First Peoples Hospital Animal Experimentation Ethics Committee.

### Cell lines, peptides and antibodies

Murine mast cell MC/9 cell line was purchased from ATCC, USA and cultured following the protocols in the product sheets. Briefly, MC/9 cells were cultured in complete RPMI 1640 media (Gibco) supplemented with 10% heat inactivated fetal calf serum (FCS), 100 U of penicillin/ml and 100 μg of streptomycin/ml and were cultured at 37°C with 5% CO_2_ and 5 ng of murine IL-4 or 1 ng of human IL-10 as recommended by ATCC, with or without adding P1, P2 or P3 respectively. MC/9 cell proliferation was determined by MTT assay (purchased from ATCC, USA) following the instruction of manufacturer.

Human macrophage cell line U937 was maintained in complete RPMI 1640 media (Gibco) supplemented with 10% heat inactivated FCS, 100 U of penicillin/ml and 100 μg of streptomycin/ml and were cultured at 37°C with 5% CO_2_.

Long HPV16 E7 peptide GQAEPDRAHYNIVTFCCKCDSTLRLCVQSTHVDIR, and HPV16 E7 CTL epitope RAHYNIVTF, Ova specific CTL epitope SIINFEKL were synthesised and purified by Mimotopes (Melbourne, Australia). Designed peptides P1, P2, P3 and P4 were synthesized by *GenicBio Biotech* (Hongkong, China). The purity of the peptides was determined by reverse-phase HPLC and was found to be more than 95%. Peptides were dissolved in 0.5% DMSO in PBS and, if not used immediately, stored at -20°C. Lipopolysaccharide (LPS) and Incomplete Freund’s adjuvant (IFA) were purchased from Sigma.

Recombinant Human interleukin 10 receptor alpha was purchased from Creative BioMart, USA (Cat. No IL10RA-212H), and was re-suspended in sterilized Milli Q water to a concentration of 1 μg/μL as stock solution. Recombinant Human interleukin 5 receptor alpha was purchased from Genscript, USA (Cat. No Z03126-10), and was re-suspended in sterilized Milli Q water to a concentration of 1 μg/μL as stock solution.

Human IL10 was purchased from ebioscience (Cat. No: BMS/346). Mouse IL4 was purchased from ebioscience (Cat. No: 14–8041).

Anti-IL10 receptor (1B1.3) monoclonal antibody (MAb) for *ex vivo* immunisation was purchased from BioXcell, USA and stored at -80°C till further use. Anti-IL-10 (Cat. 506802), Anti-IL-10R antibodies (Cat. 308802) for *in vitro* experiments were purchased from *BioLedgend*. PE conjugated anti-IL10R antibody was purchased from *BioLegend* (Cat. 308803).

### Direct binding assays

#### Surface Plasmon Resonance (SPR) Spectroscopy

The SPR assays were determined at 25°C on a Biacore T100 SPR instrument, using a CM5 sensor chip immobilized with IL-10 and IL-10R1 by GE Amine Coupling Kit. A channel treated following the same procedure but without IL-10 or IL-10R1 immobilisation was employed as a blank reference. The running buffer was 1X phosphate buffered saline at pH of 7.4. To determine the surface binding affinity, peptides diluted with the running buffer at various concentrations were injected at a flow rate of 10 μL/min for 1 min, followed by 5 min dissociation. Sensorgrams from each cycle was subtracted by the corresponding blank run. Then steady-state affinity analysis was performed using Biacore T100 Evaluation Software v2.0.3 (GE Healthcare) based on three independent repeats. For the competitive binding assay, peptides at various concentrations were co-injected with a low concentration of IL-10 (33 nM) through the IL-10R1 channel. Sensorgrams from each cycle was blank and baseline corrected, and then compared with sensorgrams with only IL-10 or the corresponding peptide at the same concentration. The inhibition of IL-10 binding to IL-10R1 was indicated by a loss in total response unit (RU).

#### MALDI (Matrix-assisted laser desorption ionisation) mass spectrometry analysis

The mass spectra of IL-10R1/peptide complexes were obtained using a 4700 MALDI-TOF/TOF mass spectrometer (*AB Sciex Pte*. *Ltd*., *USA*). The methods of using MALDI mass spectrometry to study the protein/protein and protein/peptide complexes have been reported previously[33–35]. Either 4-hydroxy-3-methoxy cinnamic acid (ferulic acid [FA], *Sigma-Aldrich*, Cat. 46278-1G-F) or 3, 5-Dimethoxy-4-hydroxycinnamic acid (sinapic acid, *Sigma-Aldrich*, Cat. 85429-1G) was employed as the matrix to obtain the optimal spectra; they were dissolved in 50% methanol/50% MQ, or 60% CAN/40% MQ/0.1% TFA, respectively, as a saturated solution. The peptides were prepared in MQ water at a concentration of 1×10^−6^ mol/mL, and IL-10R1 was reconstituted in MQ water at a concentration of 6×10^−8^ mol/mL. A sample mixture consisting of an equal volume of protein and peptide at 4×10^−8^ mol/mL was incubated on ice for 2 hours, and utilized for mass analysis. From this mixture, 0.25 μL of matrix solution was spotted first on a 192-wells plate (Cat. 4333375, *AB SCIEX* Australia Pty Ltd), and then 0.25 μL of mixture was spotted on the same plate, followed by another layer of 0.25 μL matrix solution added on to the top of air-dried sample spot prior to introduction into the mass spectrometer. The mass spectra were acquired using the linear positive ion mode of MALDI-TOF MS.

### Cell-based assays

*In vitro stimulation of U937 cells*: 2–5×10^5^ of U937 cells were cultured in 1 mL of RPMI with 10% human serum containing 100 U of ampicillin and 100 U of streptomycin. The U937 cells were either unstimulated or stimulated with 4×10^−3^ μM of LPS (Sigma, Cat. L3024-5MG) overnight, in the presence of different concentration of P1, P2 or P3 or anti-IL-10 (10 μg/mL, ~0.3 μM) or anti-IL-10R (10 μg/mL, ~0.1 μM) antibodies. Supernatants were collected and stored at -80°C till use.

*Surface staining of IL-10 receptor on U937 cell membrane*: 2–5×10^5^ of U937 cells were stained with PE conjugated anti-IL10R antibody for 30 minutes on ice, then washed with PBS with 2% of FCS and analysed with an Acuri flowcytometor (BD, USA) as described previously [36].

*Isolation of peripheral blood mononuclear cells*: Heparinised (25 IU/mL) peripheral blood was taken from informed individuals, and peripheral blood mononuclear cells (PBMCs) were separated by discontinuous density gradients of Ficoll-Hypague (GE Healthcare) according to the manufacturer's protocol. Isolated PBMCs were washed extensively using RPMI with 10% of human serum. This bioassay was carried out strictly under research ethics and approached by the Human Ethics Committee of the 1^st^ People’s Hospital of Foshan, China.

*In vitro stimulation of PBMCs*: 2–5×10^5^ of PBMCs were cultured in 1 mL of RPMI with 10% human serum containing 100 U of ampicillin and 100 U of streptomycin. The PBMCs were either unstimulated or stimulated with 4×10^−3^ μM of LPS (Sigma, Cat. L3024-5MG) overnight, in the presence of different concentration of P1, P2 or P3 or anti-IL-10 (10 μg/mL, ~0.3 μM) or anti-IL-10R (10 μg/mL, ~0.1 μM) antibodies. Supernatants were collected and stored at -80°C till use.

*7-AAD assay*: 7-AAD Viability Staining Solution was purchased from eBioscience (Cat. 00–6993). Briefly, 5 μL of 7-aad were added to 5×10^5^ cells for 5 minutes, and 7-aad staining was measured by a BD Accuri flow cytometry.

#### ELISA

Human IL-10 ELISA MAX^™^ Deluxe 5 plates were purchased from Biolegend (Cat. 430604) and performed following the manufacturer’s instruction. Human IL12p40 and IL12p70 ELISA kits were purchased from eBioscience, IL12p40 or IL12p70 levels from supernatants collected from stimulated PBMCs were measured by ELISA following the instruction from manufacturer.

### *Ex vivo* assay

#### Immunization of mice

Groups of five to eight mice were immunized s.c. with 50 μg long Human Papillomavirus Type 16 (HPV16) E7 peptide, or 15 μg of LPS, with or without 250 μg of anti-IL10R antibodies dissolved in PBS. In the case of immunization with incomplete Freund's adjuvant (IFA), the dissolved long HPV16 E7 peptide was emulsified in 50% (v/v) IFA before s.c. vaccination. The total injected volume was 100 μL/mouse. Mice were lightly anaesthetized with isofluorane (Abbott, Maidenhead, U.K.) during immunization.

#### ELISPOT

ELISPOT was performed as described previously [37]. Briefly, single spleen cell or lymph node suspensions were added to membrane base 96 well plate (Millipore, Bedford, MA) coated with anti-IFN-γ (BD Harlingen, San Diego, CA). Designed peptide was added at various concentrations and cells incubated with the peptide at 37°C for 18 hours. Antigen specific IFN-γ secreting cells were detected by sequential exposure of the plate to biotinylated anti-IFN-γ before s.c. vaccination (BD Harlingen), avidin–horseradish peroxidase (Sigma–Aldrich) and DAB (Sigma–Aldrich). The results were measured by ELISPOT reader system ELR02 (AID Autoimmun Diagnostika GmbH, Strassberg, Germany).

### Statistical analysis

Statistical analysis was performed by the two tailed Student’s test. Survival rate comparison among different groups was performed by log rank test, by using Prism 5.0 (Graphpad Software, San Diego). Results are considered as significant if P value is less than 0.05.

## Results

### The design of peptide inhibitors of IL-10

**Fig 1:**
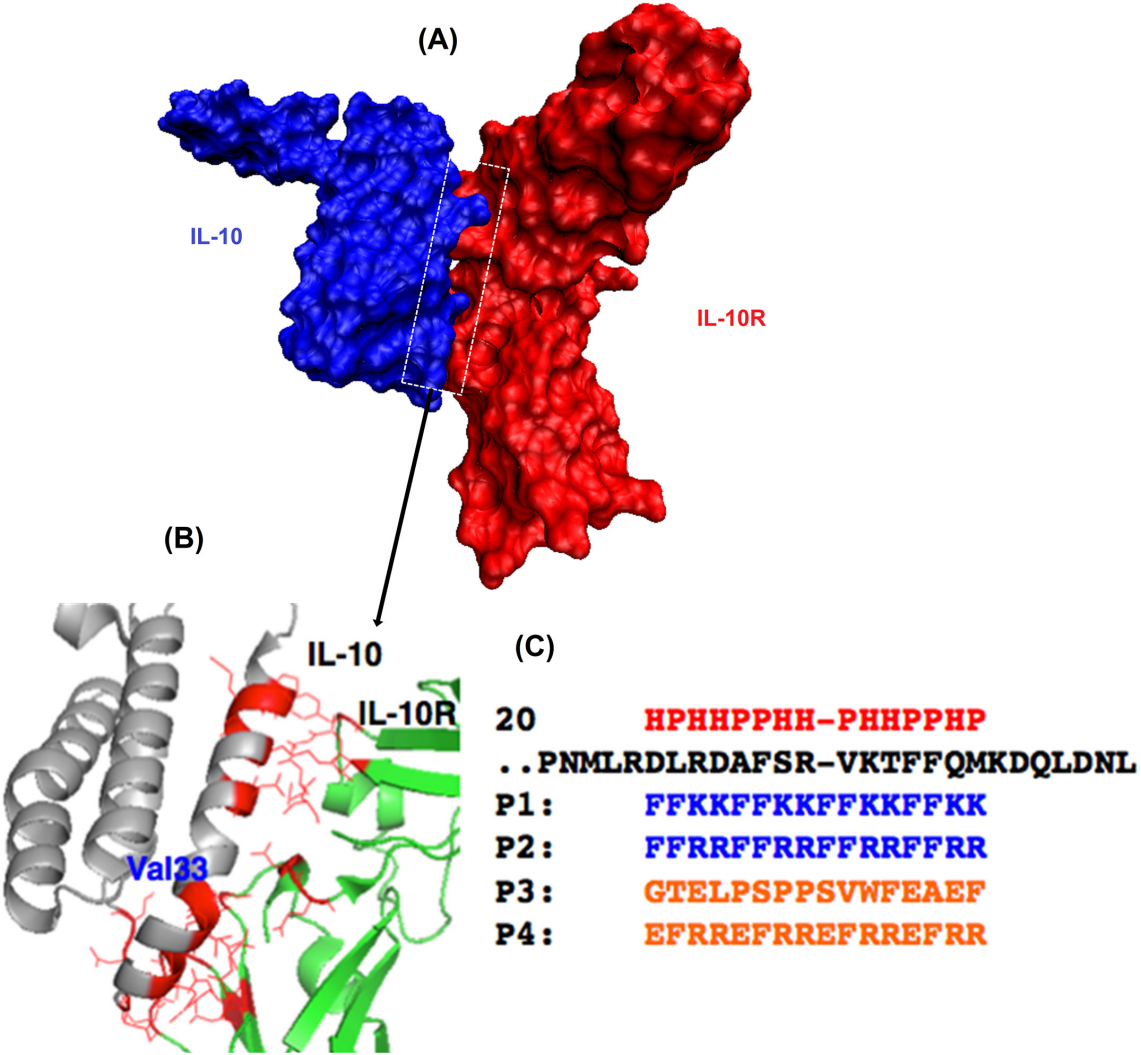
Illustration of peptide design. (A) A space-filling model of the interface structure of the IL-10/IL-10R protein–protein complex. (B) An expansion of the interface between the two proteins to indicate a few residue to residue contacts. The complex structure of IL-10 (Grey) and IL-10R1 (Green) along with the binding interface (Red). Val33 indicates the location of a helical bend for the binding helix. (C) Mimicking of this interface could produce an antagonist to inhibit the interaction. The binding helical region of IL-10 (black) is aligned with designed peptides (P1 and P2[38] in Blue) along with the hydrophilic/hydrophobic (HP) pattern (Red). Two control peptides (P3 and P4) are also shown (Orange).

The specific interaction between IL-10 and IL-10R dictates their biological events. The specificity requires the complementary in terms of their surface topology and chemical composition. As shown in Fig 1, a helical segment of IL-10 (Helix 1 of IL-10) is responsible for most binding interactions with IL10-R. In addition, there are significantly more electron negative oxygen atoms (either carboxylate or hydroxyl oxygen) than acidic hydrogens (amide hydrogen on backbone, or those on side chains of certain amino acids) in IL10-R binding residues, thus we speculated that peptide sequenced with more of acidic hydrogens would favour its interference with IL-10/IL-10R interaction. We employed the hydrophobic and hydrophilic pattern of the helix segment of IL-10 to designed peptide inhibitors. This helix ranges from residues 20 to 40 (PDB 1J7V, S1 and S2 Tables). Helix 1, however, is not straight but bends at residue Val33 (Fig 1B). Val33 is surrounded by a repeated HHPP pattern for a helical conformation (Fig 1C) where HP denotes hydrophilic and hydrophobic residues, respectively.

Thus, we designed our peptides based on this HHPP pattern but without breaking the pattern at Val33, to strengthen its helical conformation. We employed Phe (F) for representing hydrophobic residues as it has the highest number (3) of occurrence near the bend. Then, we picked positively charged Lys (K) or Arg (R) for representing hydrophilic residues and improving solubility as both appeared in the helix. The sequences of our designed peptides 1 and 2 (P1 and P2) are aligned with the binding helix in Fig 1B. As a control, we also designed a peptide P3 with no similar pattern and high ratio of electron negative residues, to make it more likely to be repellent to the binding area. Besides, a peptide P4 with a HPHH pattern was obtained as a second control.

### Binding of P1 and P2 to IL-10 and IL-10R1

**Fig 2:**
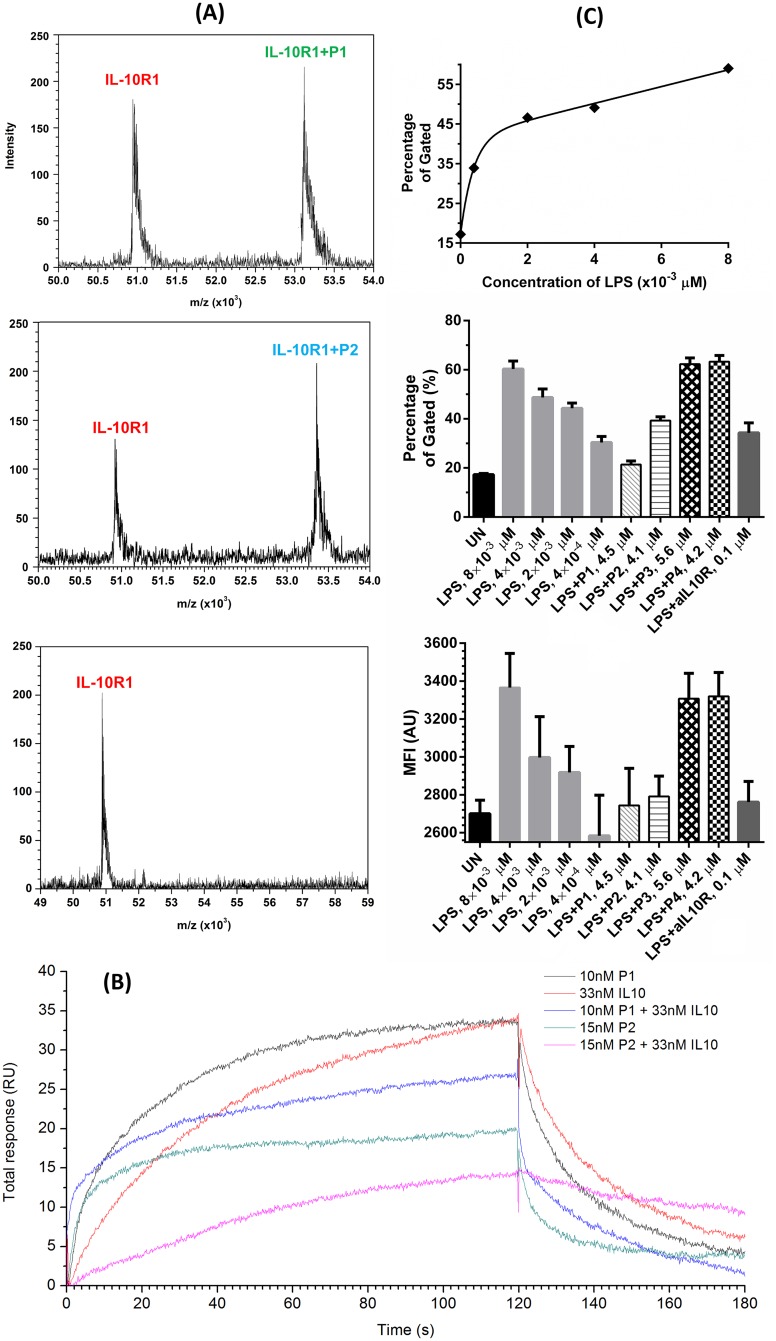
(A) MALDI mass spectra of IL-10R1 when mixed with P1, P2 and P3 as shown. Only P1 and P2 displayed a peak corresponding to the mass of the complex structure. (B) An overlay of sensorgrams of the SPR competitive binding assay of peptides P1 and P2. Compared with sensorgrams with only IL-10 or the corresponding peptide at the same concentration, a loss in total response was observed when P1 or P2 were co-injected with IL-10. (C) IL-10R expression levels in 3×10^5^ U937 with anti-human CD210 using flow cytometry: stimulated with different amount of LPS overnight; unstimulated and stimulated with LPS (4×10^−3^ μM), LPS (4×10^−3^ μM) + P1 (4.5 μM), P2 (4.1 μM), P3 (5.6 μM) or P4 (4.2 μM), and LPS (4×10^−3^ μM) + aIL10R1 overnight, respectively; the mean fluorescence intensity (MFI) result of IL10-R expression.

First, we obtained mass spectra of IL-10R1 mixed with designed peptides P1 and P2 and the random control peptide P3. As Fig 2A shows, only P1 and P2 form a complex structure with IL-10R1 detectable by MALDI MS using linear mode under the condition. As they were designed to mimic the binding between IL-10 and IL-10R1, we further confirmed that P1 and P2 bind with IL-10 as well by the MALDI MS. As shown in S1 Fig, both P1 and P2 but not P4 oligomerised with IL-10, and they formed multiple charged complexes with different molar ratios, including 4:1, 5:4 and 5:3 (IL10-R1/peptide). To address the specificity of the interaction, the possibility of the interaction of P1 and P2 with another cytokine IL-4 and cytokine receptor IL-5R, was also examined using MALDI MS. No peak corresponding to any complex was observed within a broader mass range (10 to 100 kD), when P1 or P2 was mixed with IL-4 or IL-5R (S2 and S3 Figs). P4 did not form any detectable complexes with any of these proteins, though it would self-oligemerise with the presence of IL-5R (S3C Fig), a similar scenario also occurred in the case of P2 plus IL-5R.

Next, we performed binding affinity analysis by SPR spectroscopy (Fig 2B). P1 and P2 displayed a level of binding affinity with IL-10R1 at 16.0 ± 1.95 μM and 62.1 ± 8.84 μM, respectively and with IL-10 at 8.94 ± 0.37 μM and 36.1 ± 2.40 μM, respectively. No binding to IL-10R1 or IL-10 was observed for either P3 or P4. We also observed that 10 nM of P1 or 15 nM of P2 can prevent 33 nM of IL10 from binding to immobilised IL10-R1, resulting in a lower total response unit compared with only the peptides or IL-10 at the corresponding concentrations. No such competitive binding was observed for either P3 or P4.

Additionally, we investigated the intensity of anti-IL-10R antibody binding to IL-10R on the membrane of human macrophage U937 cells by using a PE conjugated anti-IL-10R antibody (clone: 3F9) and analysed the intensity of this antibody binding to IL-10R1 by flow cytometer. More antibody binds to IL-10R1 after LPS stimulation, and is positively correlated with the dose of LPS (Fig 2C). Binding to IL-10R was reduced when either P1 or P2, but not P3 or P4 was present in culture of LPS stimulated U937 cells, suggesting P1 and P2 compete with anti-IL10R antibody in binding to IL-10R (Fig 2C).

### P1 and P2 prevent proliferation of IL-10 dependent MC/9 cell line

**Fig 3:**
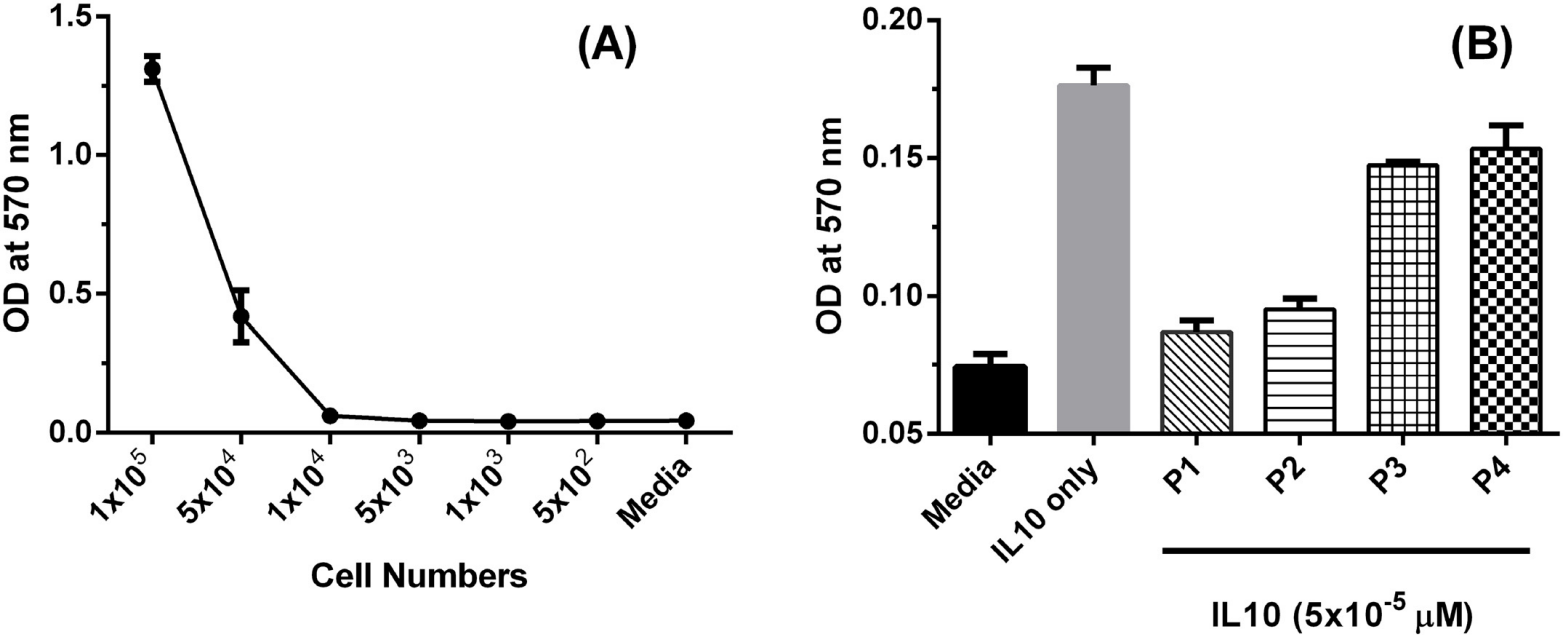
P1 and P2 are able to inhibit growth of mouse MC/9 mast cells measured by MTT assay. (A) Different numbers of mouse MC/9 mast cells were cultured in RPMI 1640 media with 10% FCS and 2×10^−5^ μM of IL4 for 48 hours, blue arrow indicated the cell numbers chosen for maximal growth potential. (B) 1×10^4^ of MC/9 cells were cultured either in media with 2×10^−5^ μM of IL4 (media), or plus 5×10^−5^ μM of IL10 (IL10 only), or plus 5×10^−5^ μM of IL10 together with different peptides (P1, P2, P3 and P4 at 10 μg/mL, equals to 4.5, 4.1, 5.6 and 4.2 μM, respectively) for 48 hours before MTT assay was performed.

Mouse mast cell line MC/9 growth is IL-10 dependent [39]. We therefore investigated whether P1 or P2 is able to prevent IL-10 mediated MC/9 cell growth. MC/9 cells were cultured at different numbers for 48 hours and cell proliferation was measured by MTT assay, which measures the MC/9 cell proliferation. MC/9 proliferation showed significant increment and can be easily detected when 1×10^4^ of MC/9 cells or more cells were cultured 48 hours for MTT assay (Fig 3A). Thus, cell number of 1×10^4^ was selected in the assay. The addition of 5×10^−5^ μM of IL-10 (the dose used in IL10 dependent MC/9 cell culture [39]) led to approximately 133% increase in cell proliferation (Fig 3B). This IL-10 promoted cell proliferation was inhibited effectively by adding either P1 or P2, showing slight higher level compared to that with only culture media, but not by P3 (5.6 μM) or P4 (4.2 μM), respectively (Fig 3B). It seems the presence of P1 or P2 counteracted the effect of IL-10, suggesting that the IL-10 was inhibited by P1 or P2, but not P3 and P4.

### P1 and P2 inhibit LPS-mediated IL-10 production by human macrophage U937 cells

**Fig 4:**
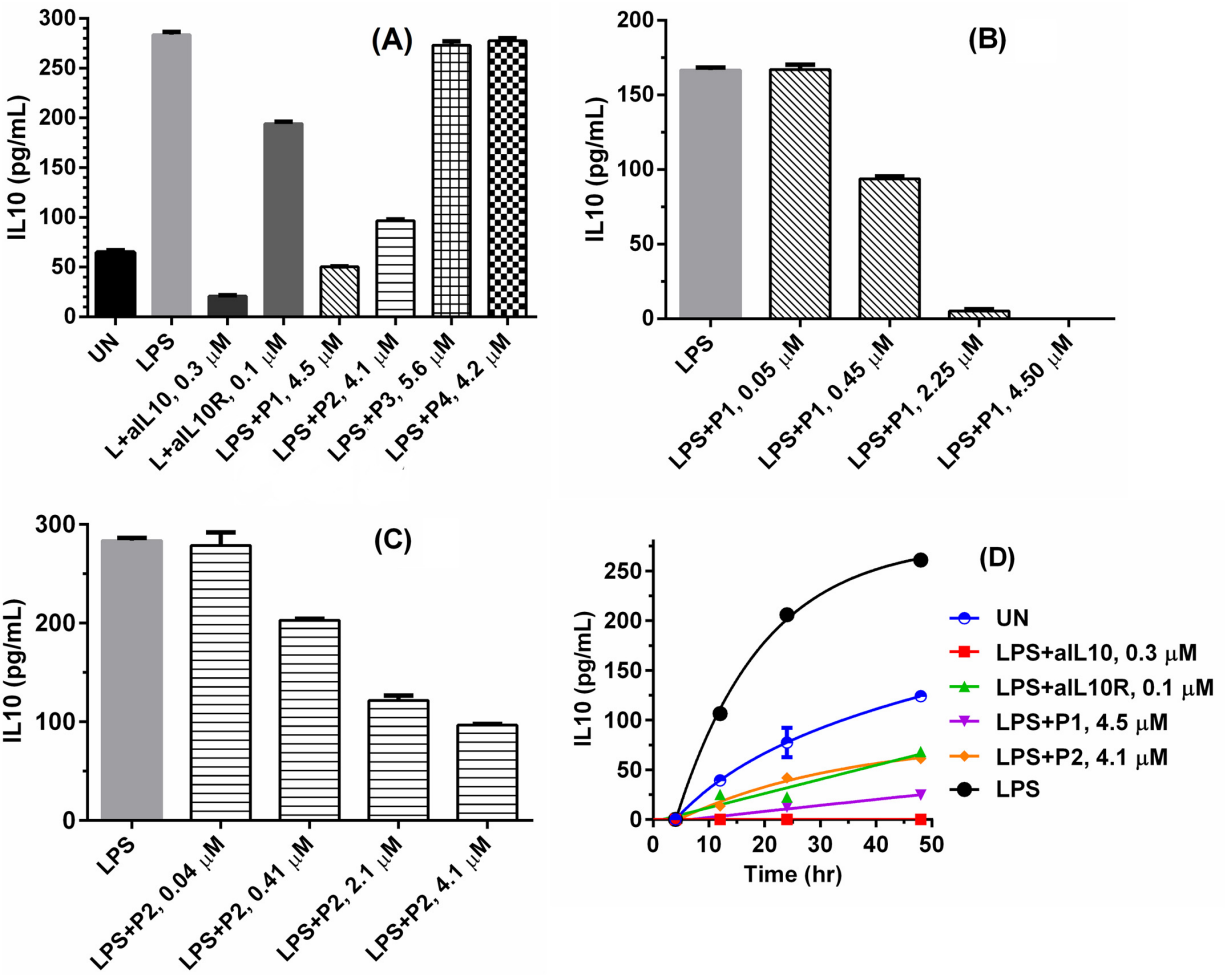
P1 and P2 reduce IL-10 secretion in U937 cells stimulated by LPS. Supernatants were measured in the presence of IL-10 by ELISA. The concentration of LPS is 4×10^−3^ μM: (A) 3×10^5^ human U937 were either left unstimulated (UN) or stimulated with LPS, LPS+0.3 μM of anti-IL10 (aIL10), LPS+0.1 μM of aIL10R, LPS+P1, 2, 3 and 4 at 4.5, 4.1, 5.6, 4.2 μM overnight, respectively. (B) 1×10^5^ of U937 cells were treated with LPS and different concentration of P1 overnight. (C) 1×10^5^ of U937 cells were treated with LPS and different concentration of P2 overnight. (D) 1×10^5^ of U937 cells were unstimulated or stimulated with LPS, LPS+aIL10, LPS+aIL10R, LPS+P1 and LPS+P2 for 4, 12, 24 and 48 hours.

The inhibition of IL-10 signalling by P1 and P2 was further studied in biological assays by employing a macrophage U937 cell line and human peripheral blood mononuclear cells (PBMCs). U937 and PBMCs secrete IL-10 when stimulated with Toll like receptor ligands CpG or lipopolysaccharide (LPS) [40], to maintain a balanced immune response. The secretion of IL-10 is reduced in the presence of anti-IL10 or anti-IL10R antibodies, while the production of IL-12 increased [41, 42]. We first reproduced these trends (S4 Fig) and then showed that P1 and P2 exhibited a similar effect of anti-IL-10 or anti-IL-10R antibodies (Fig 4A) that they decreased the level of IL-10 by approximately 80% (P1 at 4.5 μM) and 64% (P1 at 4.1 μM), respectively; nor did P3 or P4 showed such activity. That was at the dose of 10 μg/mL, both P1 and P2, although not as potent as anti-IL10, can inhibit IL-10 level better than anti-IL10R (approximately 4 and 2 folds for P1 and P2, respectively). In addition, it can be seen that P1 even statistically reduced the level of IL-10 within original U937 cells before LPS stimulation, an effect similar to that of anti-IL10. The dose-dependent study (Fig 4B and 4C) showed that 50% reduction of IL-10 level required about 0.45 μM for P1 and 2.05 μM for P2, indicating the inhibitory activity of P1 was higher than that of P2. The time dependence of IL-10 secretion with and without P1/P2 inhibition suggested the sustained activity of P1 and P2 for 48 hours (Fig 4D).

### P2 increases LPS-mediated IL-12 production by human PBMCs

**Fig 5:**
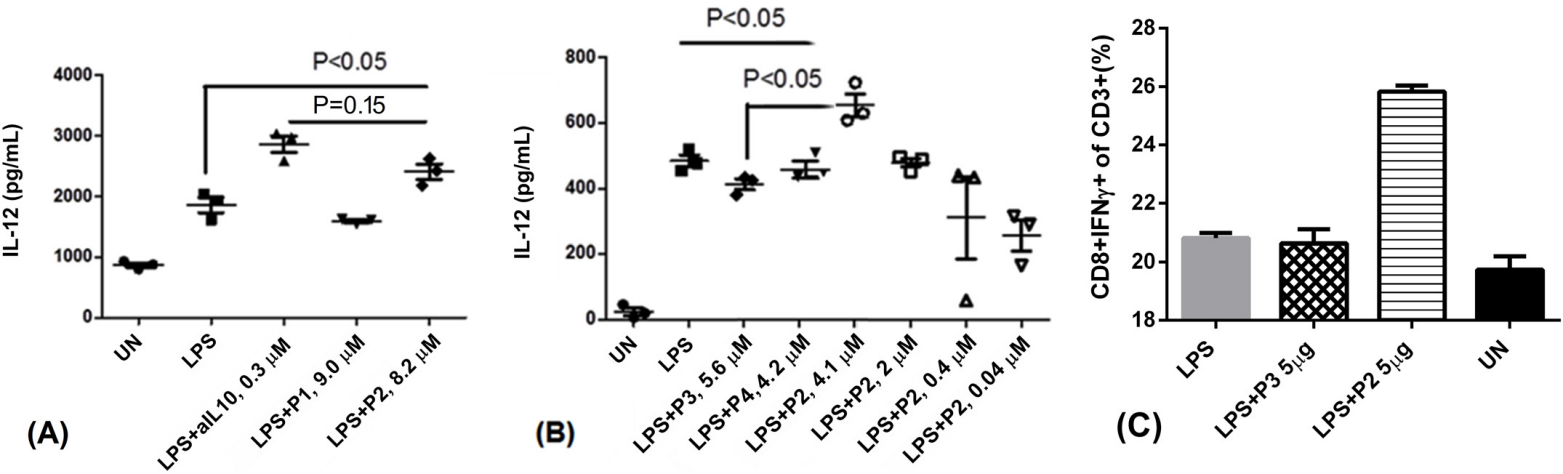
P2 increases IL-12 secretion by LPS stimulated PBMCs: (A) 3×10^5^ human PBMCs were either left overnight unstimulated (UN) or stimulated with LPS (4×10^−3^ μM), LPS+0.3 μM of anti-IL10 (LPS+aIL10), LPS and P1 at 9.0 μM, LPS and P2 at 8.2 μM, respectively. Supernatants were measured for the presence of IL-12p40 by ELISA. (B) 1×10^5^ of human PBMCs were either unstimulated or stimulated with LPS or LPS with P3, P4 and different concentration of P2 overnight, IL-12 p40 from supernatants were measured by ELISA. (C) 1×10^6^ PBMCs were stimulated in the presence of 100 ng/mL of LPS, 5 μg/mL of P2 or P3 for 72 hours. Cells were stimulated with stimulation cocktail (ebioscience) in the final 6 hours and stained with anti-CD3, anti-CD8 and intracellularly stained with IFNγ. FACS were performed and CD3+CD8+IFNγ+ were analyzed with Flowjo.

Human PBMCs secrete IL-12 upon stimulation with LPS or CpG [43], a cytokine that is able to promote Th1 immune responses for clearance of viral infected cells or tumour cells. IL-12 expression is suppressed by IL-10, and the inhibition of IL-10 should increase the production of IL-12. We thus examined IL-12p40 by ELISA. As shown in Fig 5, adding anti-IL-10 or anti-IL-10R antibodies increases IL-12 production by LPS stimulated human PBMCs. IL-12p40 (Fig 5A) production increased by about 25% in the presence of P2 at 8.2 μM, but not P1 at 9 μM. Fig 5B further confirmed that P3 or P4 didn’t increase the level of IL-12. IL-12p40 by LPS stimulated PBMCs increased as P2 concentration increasing (Fig 5B), showing a dose dependent trend. This occured despite that P1 can block IL-10 signalling more efficiently than P2 suggested by other assays (Figs 2, 3 and 4). We also found that P2 was able to promote human T cell secreting IFNγ about 5% and 6% higher than LPS stimulated and unstimulated PBMCs, respectively, which isanother evidence showing that P2 is able to promote Th1 response (Fig 5C).

### P2 increases antigen specific CD8+ T cell response induced by long E7 peptide/IFA from a human papillomavirus type 16 (HPV 16)

**Fig 6:**
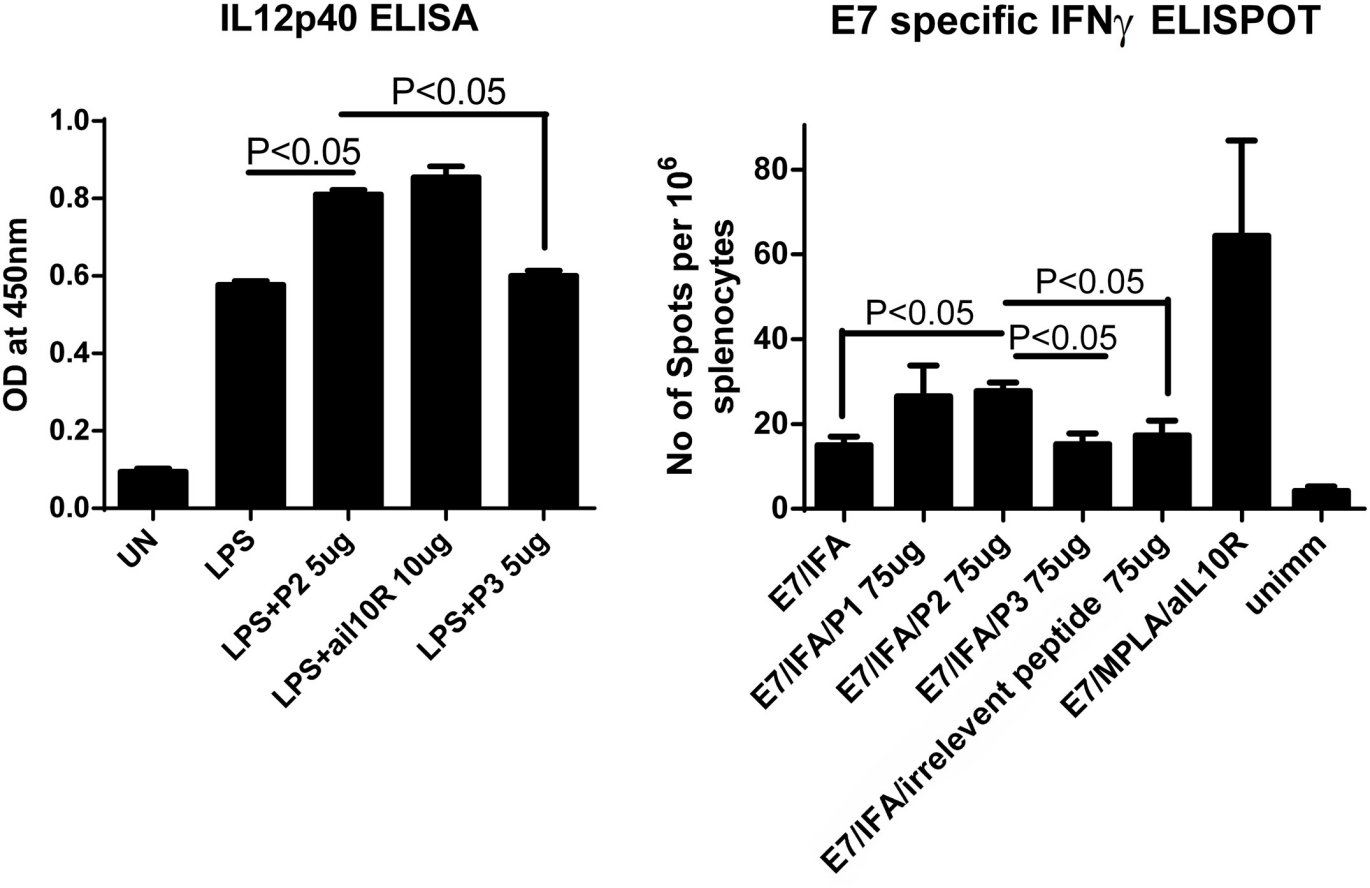
(A) 5x10^5^ of mouse splenic cells were either left unstimulated, or stimulated with 100 ng of LPS, or same amount of LPS and P2, P3 and anti-IL-10R antibodies overnight. IL12p40 from supernatants were measured by ELISA as described in Materials and Methods. (B) Four C57BL/6 mice from each group were primed either with HPV16E7 peptide/MPLA/aIL10R antibodies or with HPV16E7/IFA with P1, P2, P3, irrelevant peptide (OVA CTL epitope) or left unimmunized on day 0, and then boost immunized with long E7 peptide/IFA on day 7. Six days after final immunization, spleen from immunized mice were collected, single spleen cells isolated, and cultured in the presence of a MHC I restricted HPV16 E7 specific peptide RAHYNIVTF overnight, ELISPOT assay for IFNγ was performed as described in materials and methods.

First, mouse splenic cells were stimulated with LPS overnight, with P1, P2, P3 or anti-IL-10R antibodies respectively. IL-12p40 from supernatants was measured by ELISA. The statistical analysis showed that P2, but not P1 or P3, was able to increase IL-12 secretion by LPS stimulated mouse splenic cells, which was an effect similar to that induced by antiIL-10R antibodies (Fig 6A). It has been widely accepted that HPV 16 E7-specific cytotoxic T lymphocytes (CTLs) can be generated by immunization with E7 protein in IFA, resulting in long-lasting protection against HPV16-transformed tumor cells [44–47]. To test whether P1 and P2 can increase the CTLs, groups of mice were immunized with a long papillomavirus E7 peptide in IFA, or E7/IFA/P1, E7/IFA/P2, E7/IFA/P3, E7/IFA/OVA CTL epitope (an irrelevant peptide to HPV16), E7/MPLA/anti-IL10R antibody twice or left unimmunized at 7 days apart. Seven days after final immunization, E7 specific IFNγ CD8+ T cell responses were measured by ELISPOT assay. E7/IFA/P2 induced higher numbers of CD8 E7 specific T cell responses than E7/IFA, E7/IFA/P3 and E7/IFA/OVA CTL epitope. The result indicates that P2 was biofunctional *ex vivo* and could enhance antigen specific CD8+ T cell response induced by long E7 peptide/IFA of the human papillomavirus type 16 at a magnitude less than E7/MPLA/anti-IL10R (Fig 6B).

## Discussion

Receptor engagement with IL-10 favours the exposure of the chemotactic (-DLRDAFSRVKTFFQM-) with a helical conformation, which we chose as the key binding motif. Based on its major residue pattern of ‘HHPP’ (Fig 1), peptides (P1 and P2) were designed, which also align well with the α-helical protein folding principle:- the peptide carbonyl O atom and amide proton between the i^th^ and (i+4)^th^ amino acid positions form a paired hydrogen bonding, resulting in a folded structure with a regular turn every 3.6 amino acids [48]. It has been shown that hydrophobic interactions between the amino acid side groups contribute significantly towards the nucleation of helical conformation [49], thus Phe, an amino acid with strong hydrophobic side group, is used in P1 and P2. In addition, hydrophobic interactions between the side groups of the i^th^ and (i+4)^th^ amino acids might enhance the α helice [50]. Therefore, to maintain the α-helical periodicity, a repeat primary sequence containing 2 Phe and 2 hydrophilic amino acids is proposed. Considering there are more electron negative oxygen atoms than acidic hydrogens in IL10-R1 in the binding interface area, positive charged amino acids were chosen, i.e., Lys and Arg in the cases of P1 and P2, respectively.

Various *in vitro* studies indicated that the designed peptides, P1 and P2, could be biological active through binding to IL-10 and IL-10R1 and with a competitive binding to IL-10R1. We further showed that both P1 and P2 can inhibit IL-10 dependent MC/9 cell proliferation (Fig 3). In the presence of IL-4, IL-10 significantly promotes MC/9 proliferation [51]; when either P1 or P2 is present, this IL-10 mediated effect on MC/9 proliferation is abolished, while control peptides (P3 and P4) have no effect, suggesting that IL-10 might be inhibited by P1 or P2. P1 and P2 reduce LPS mediated IL10 secretion by human macrophage U937 cells in a dose and time dependent manner. P2 could further increase LPS induced IL-12 secretion by human PBMCs. More importantly, P2 enhances antigen specific IFNγ secreting CD8+ T cell responses induced by early protein E7 long peptide/LPS vaccination of a papillomavirus type 16 in an *ex vivo* assay.

In order for a peptide to be useful as potential drug candidates, its cytotoxicity has to be low. We evaluated the cytotoxicity of P1 and P2 using 7-AAD cell-viability assay (S5 Fig). The results of two independent assays indicated that there is no significant difference for cell death in the presence or absence of P1 or P2. That is, the cytotoxicity of P1 or P2 is negligible, indicating that the inhibition effect on IL-10 in U937 cells by P1 and P2 is not caused by cell death.

It is noted that the ELISA shows that P1 and P2 are able to reduce IL-10 secreted by U937 cells at levels much lower than the results obtained through the SPR assay, this may be attributed to the fact that P1 and P2 can also bind to IL10 as indicated by MALDI MS (S1 Fig) and SPR experiments (Fig 2B). In addition, the detection sensitivities of ELISA and SPR under different biological conditions are not always consistent [52–54]. Mullenix compared ELISA and SPR for assessing clinical immunogenicity of panitumumab, and found that ELISA was much more sensitive in detecting mAbs with high affinity than SPR due to the binding kinetics; for instance, ELISA showed a sensitivity of 0.016 μg/mL compared to 15.382 μg/mL provided by SPR for one mAb [54].

When antigen presenting cells are stimulated with Toll like receptor ligands, such as TLR4 ligand LPS, they produce both IL-10 and IL-12 [55–58]; as the secretion of IL-10 after TLRs stimulation is a feedback mechanism to prevent excess immune responses to avoid autoimmunity [59, 60]. Reducing IL-10 expression level enhances IL-12 production by antigen presenting cells, which is critical for vaccine mediated Th1 responses [43, 61, 62]. In another experiment, human macrophage cell line U937 cells were stimulated with LPS, IL-10 secretion by U937 was measured by ELISA. Blocking IL-10 signalling by adding anti-IL10, or anti-IL10R antibodies reduces IL-10 secretion by LPS stimulated U937 cells (Fig 4). Addition of P1 and P2, but not the control peptide P3 or P4 to U937 culture media, inhibits IL-10 secretion by the LPS stimulated U937 cells (Fig 4). The inhibition is not because that P1 and P2 are toxic to the U937 cells (S5 Fig). All of these *in vitro* cell-based assays suggest that the designed peptide successfully inhibit the intended target IL-10.

Because P2 can enhance IL-12 secretion by LPS stimulated human PBMCs *in vitro* (Fig 5), we investigated whether P2 is bioactive *ex vivo* by using a mouse vaccination model based on early protein 7 (E7) long peptide/IFA of human papillomavirus type 16 [63]. We found that P2 not only enhances IL-12 production by LPS stimulated mouse splenic cells (Fig 6A), but also increases vaccine-induced, antigen-specific CD8+ T cell responses (Fig 6B). These results indicate that P2 is also bioactive *ex vivo*. Taken together, we have designed potential IL-10 inhibiting peptides by using a structure-based method. The reason why P1 cannot enhance the secretion of IL-12 remains elusive; it may be attributed to the interplay between P1 and other cellular factor(s) on IL-12 pathways, which opens for further investigation.

IL-10 inhibition clears chronic viral infection in animal infection models [64–67]. Inhibiting IL-10 at the time of immunization enhances T cell responses induced by peptide [63], virus like particle [37], DNA [68], adenovirus [69] vaccines. Immunization together with IL-10 inhibition further improves clearance of chronic viral [68], bacterial [70] infection and prevent tumour growth [63]. As a result, many attempts have been made to design IL-10 inhibitors that may be clinically useful. Recombinant or phage display techniques have been employed for identifying protein or peptide based IL-10 inhibitor, but they are labour intensive and time consuming [55, 71, 72]. Although peptides screened by phage display bind to IL-10 receptor at lower concentrations than our designed peptide, structure-based design provides an inexpensive alternative.

Therapeutic vaccines have begun to show efficacy against Human papillomavirus infection that is related pre-cancers [73–76]. The efficacy of 16 E7 long peptide/IFA based vaccine is demonstrated in a double blind placebo controlled trial [75, 76]. However, the efficacy of the same vaccine remain to be established in cervical cancer patients [73]. Combining therapeutic vaccine with other treatment, and further increasing the vaccine induced responses are highly desirable [73]. Long E7 peptide/IFA combined with a IL-10 peptide inhibitor enhances vaccine-induced, antigen-specific CD8+ T cell response (Fig 6). Our unpublished data also indicate that inhibiting IL-10 at the time of immunization does not lead to unwanted side effects in important organs include intestine, where IL-10 knockout mice develops chronic inflammation [60]. These results argue strongly to move our current vaccination strategy from bench side to clinical trial.

## Conclusions

We reported here a structure-based design for discovering new protein topo-mimetics that inhibits IL-10/IL-10R interaction. We demonstrated that designed peptides P1 and P2, but not control peptides, bind to IL-10R1 by different *in vitro* assays. P1 and P2 inhibit the growth of IL-10 depending murine mast cell MC/9, as well as LPS mediated IL-10 production by human macrophage cell line U937, in a dose and time dependent manner. P2 also promotes LPS mediated IL-12 production by human PBMCs. Moreover, P2 enhances CD8 T cell responses induced by an E7 peptide based vaccine for human papillomavirus 16.

As a proof of concept, in this work, we used the *in vitro* and *ex vivo* assays to show that this particular α-helix segment with “HHPP” pattern could be targeted to design IL-10 inhibiting peptide. The designed sequences worth further validation and optimisation. Other peptide segments of IL-10 or IL-10R might also be used to direct the design of inhibitors, however, the determination of the key structural characteristic would be vital. In a broader context, the results from this study provide ideas into the development of much simpler peptide-based therapeutics than the resource-consuming techniques such phage display and humanized monoclonal antibody production. Since more disease-related proteins and receptors have been characterised with reliable and generic structural information, this design concept could be expanded and utilised in the design of protein surface topo-mimetics for a similar scenario.

## Supporting Information

S1 Fig MALDI mass spectra of IL-10 mixing with P1, P2 and P4: (A) IL-10, (B) IL-10+P1, (C) IL-10+P2 and (D) IL-10+P4. Only P1 and P2 display peaks corresponding to the mass of the peptide-protein complex structures (in the spectra, X = IL-10, B = P1 and C = P2).
(TIF)

S2 Fig MALDI mass spectra of IL-4 mixing with P1, P2 and P4: (A) IL-4, (B) IL-4+P1, (C) IL-4+P2 and (D) IL-4+P4. No peak corresponding to the mass of the peptide-protein complex structure was found. Only peaks of IL-4 oligomers can be observed (e.g., 2^1+^ denotes to singly charged dimer).
(TIF)

S3 Fig MALDI mass spectra of IL-5R mixing with P1, P2 and P4: (A) IL-5R, (B) IL-5R+P1, (C) IL-5R+P2 and (D) IL-5R+P4. No peak corresponding to the mass of the protein-peptide complex structure was found (in the spectra, X, B and C denote to IL-10, P2 and P4, respectively). P2 and P4 would oligomerise under the condition, respectively.
(TIF)

S4 Fig Anti-IL10 (aIL10) and anti-IL10R (aIL10R) reduce IL-10 secretion by LPS stimulated U937 cell. Supernatants were measured for the presence of IL-10 by ELISA. The amount of LPS (abbreviated as ‘L’ when coupled with other reagents) is 4×10^−3^ μM, 3×10^5^ human U937 cells were either left unstimulated (UN, repeated) or stimulated with LPS (repeated), LPS+aIL10 with different concentration, LPS+aIL10R with different concentration overnight, respectively.
(TIF)

S5 Fig Two independent 7-AAD cell death assays measured by flow cytometry. M2 of the X axis is the 7 aad+ cells (dead cells), Y axis represents the cell numbers.
(TIF)

S1 Table Hydrogen bonds between IL-10 and IL-10R1 complex [side chain NH (H_N_), hydroxyl hydrogen (H_O_); backbone amide nitrogen (H), backbone carbonyl oxygen (O), and carboxylate oxygen (O_X_), sequences followed the PDB entry 1J7V]. (DOCX)

S2 Table Hydrophobic interaction regions between IL-10 and IL-10R1. (DOCX)
