# Supplementary figures and images for: Investigation the Possibility of Using Peptides with a Helical Repeating Pattern of Hydro-Phobic and Hydrophilic Residues to Inhibit IL-10
Source: PLoS One. 2016 Apr 21;11(4):e0153939. doi: 10.1371/journal.pone.0153939 (PMC4839630; doi:10.1371/journal.pone.0153939)

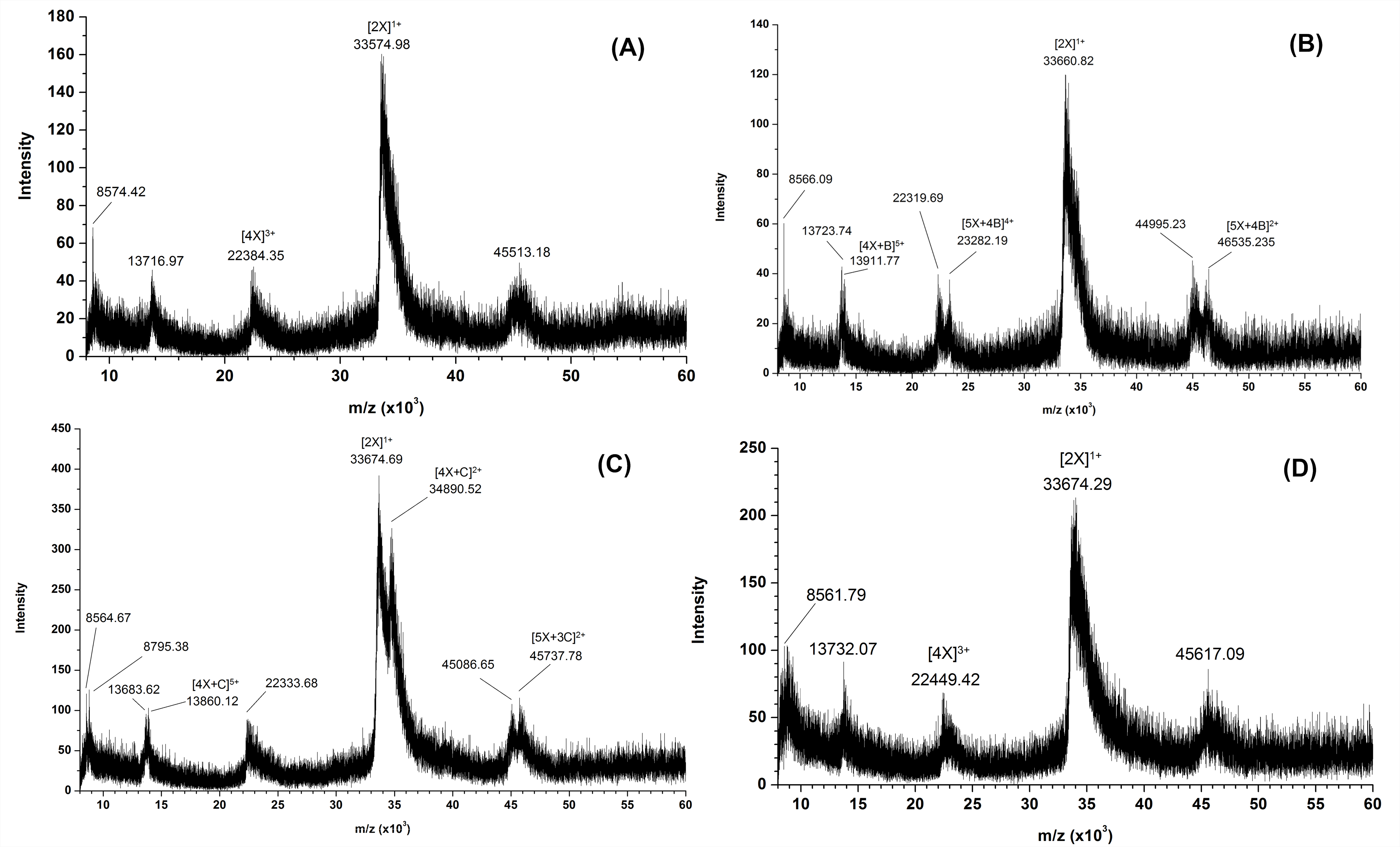

Supplement: S1 Fig — Only P1 and P2 display peaks corresponding to the mass of the peptide-protein complex structures (in the spectra, X = IL-10, B = P1 and C = P2). (TIF) [file pone.0153939.s001.tif]

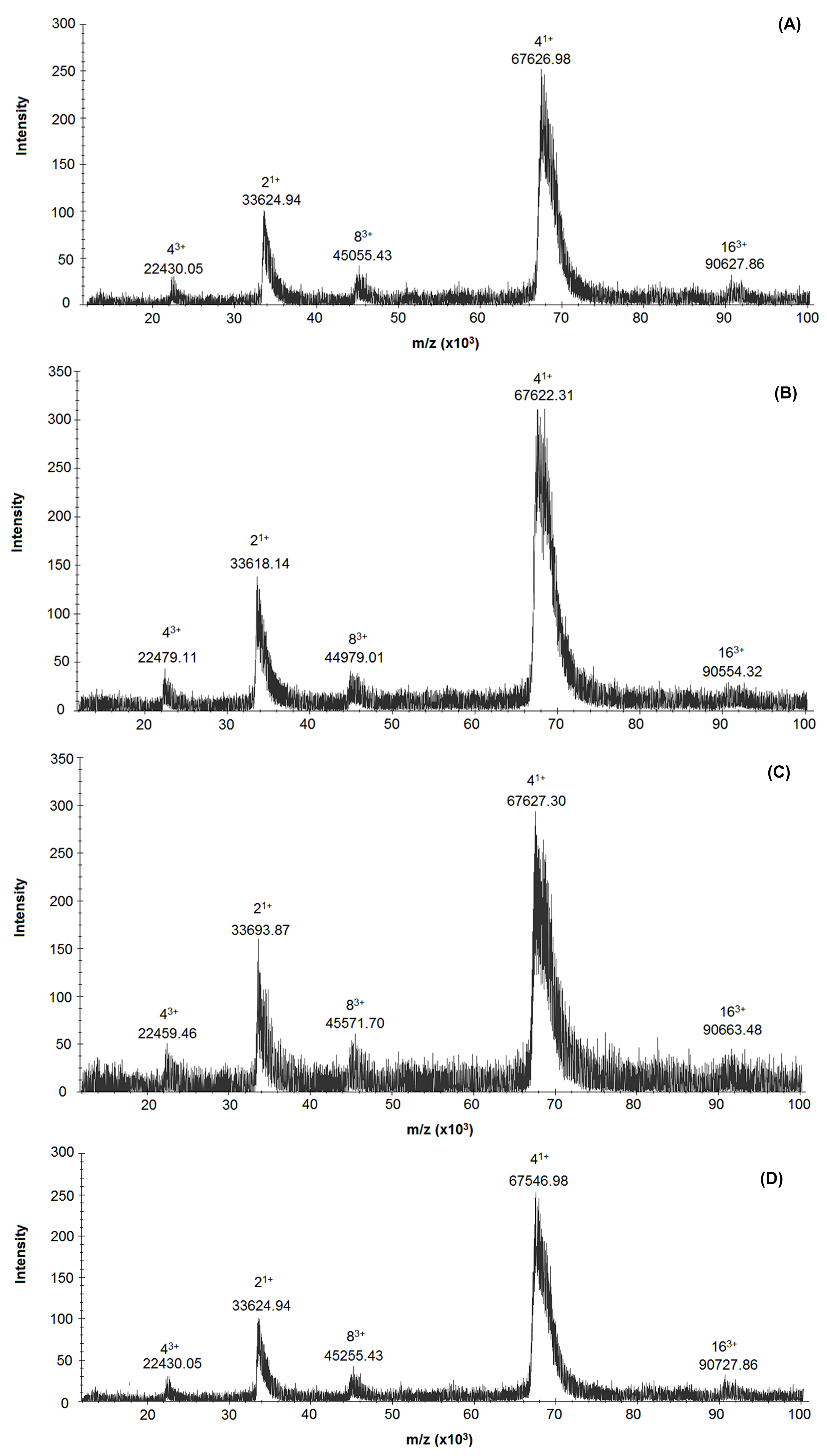

Supplement: S2 Fig — No peak corresponding to the mass of the peptide-protein complex structure was found. Only peaks of IL-4 oligomers can be observed (e.g., 21+ denotes to singly charged dimer). (TIF) [file pone.0153939.s002.tif]

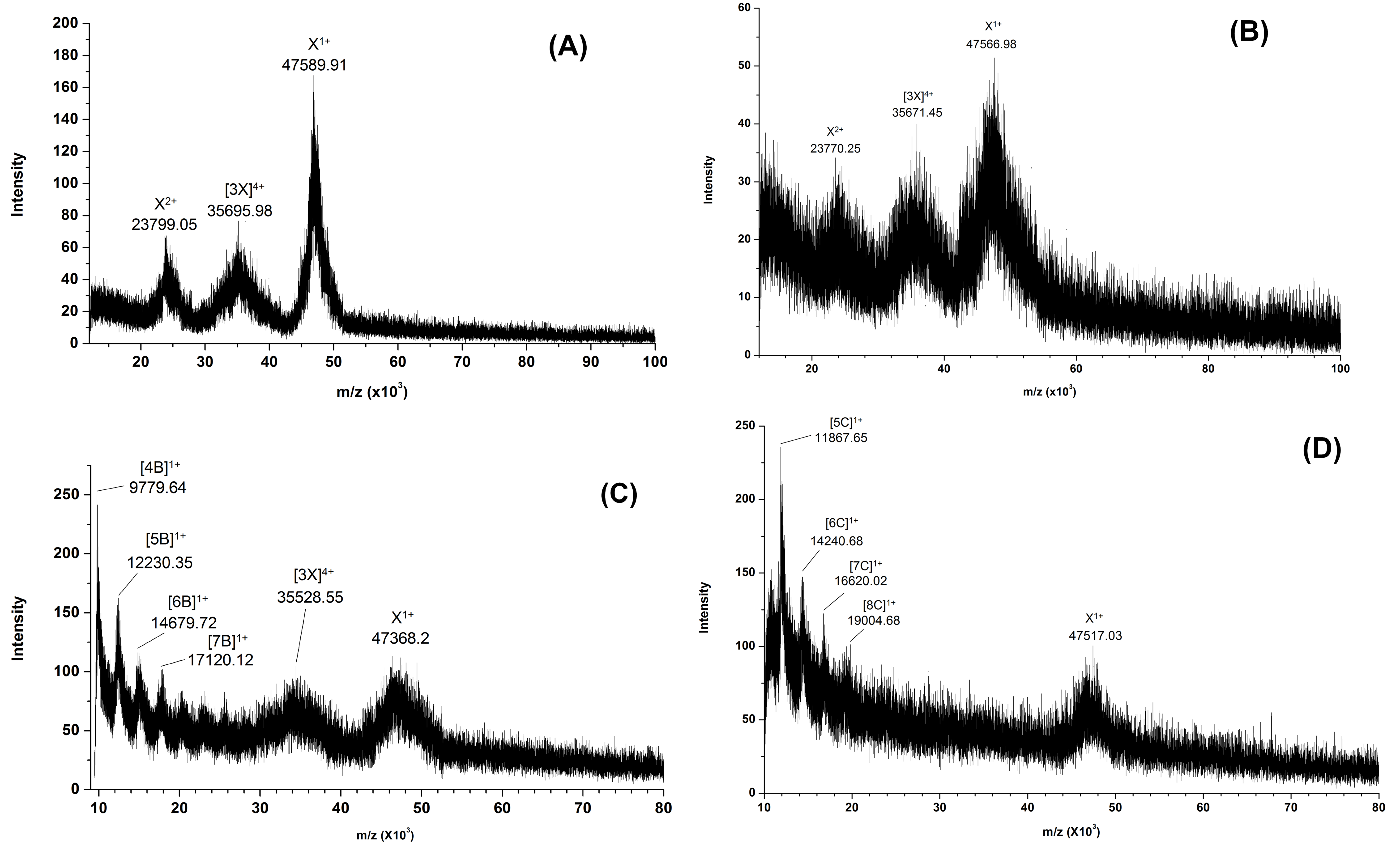

Supplement: S3 Fig — No peak corresponding to the mass of the protein-peptide complex structure was found (in the spectra, X, B and C denote to IL-10, P2 and P4, respectively). P2 and P4 would oligomerise under the condition, respectively. (TIF) [file pone.0153939.s003.tif]

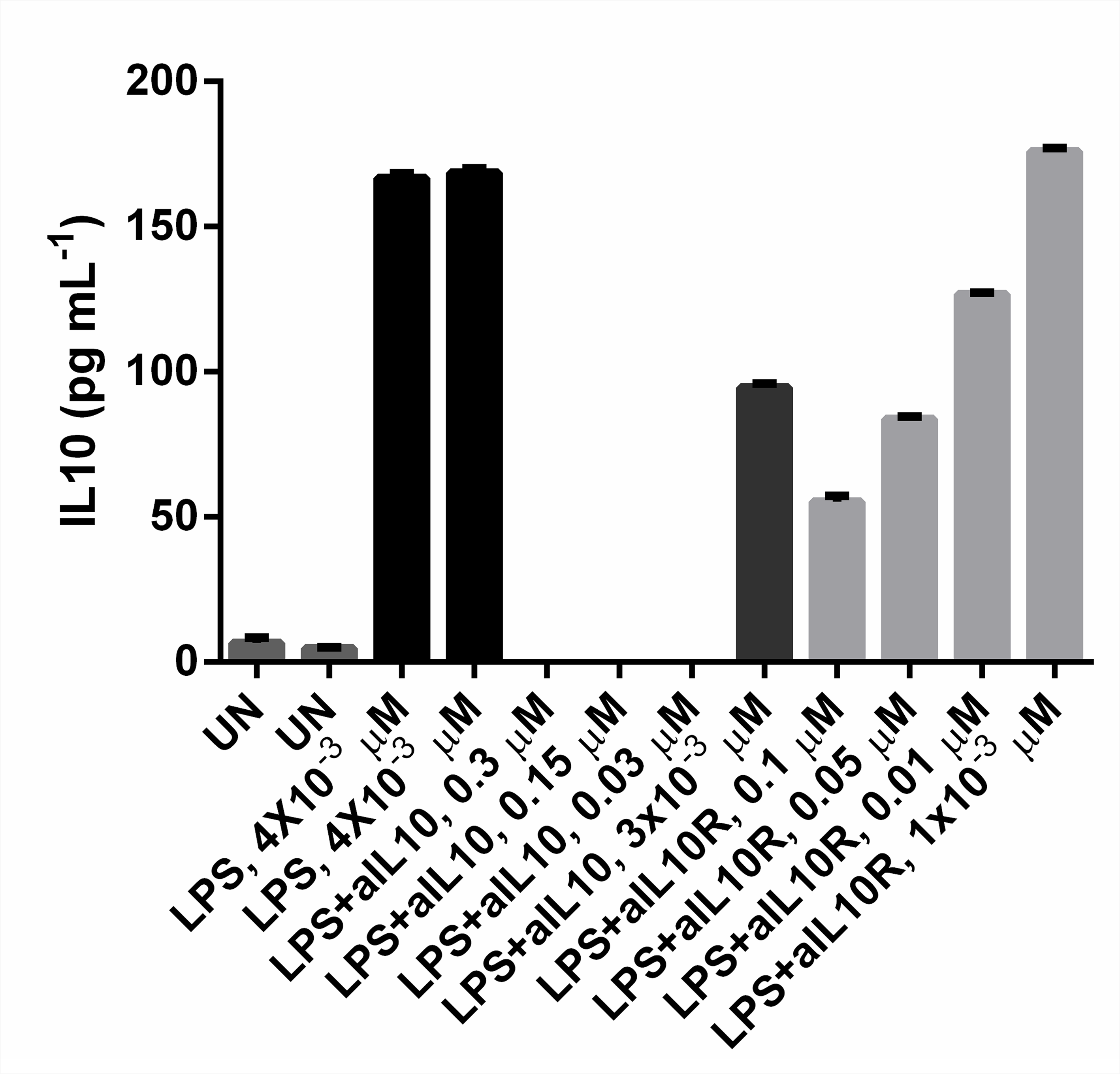

Supplement: S4 Fig — Supernatants were measured for the presence of IL-10 by ELISA. The amount of LPS (abbreviated as ‘L’ when coupled with other reagents) is 4×10^−3^ μM, 3×10^5^ human U937 cells were either left unstimulated (UN, repeated) or stimulated with LPS (repeated), LPS+aIL10 with different concentration, LPS+aIL10R with different concentration overnight, respectively. (TIF) [file pone.0153939.s004.tif]

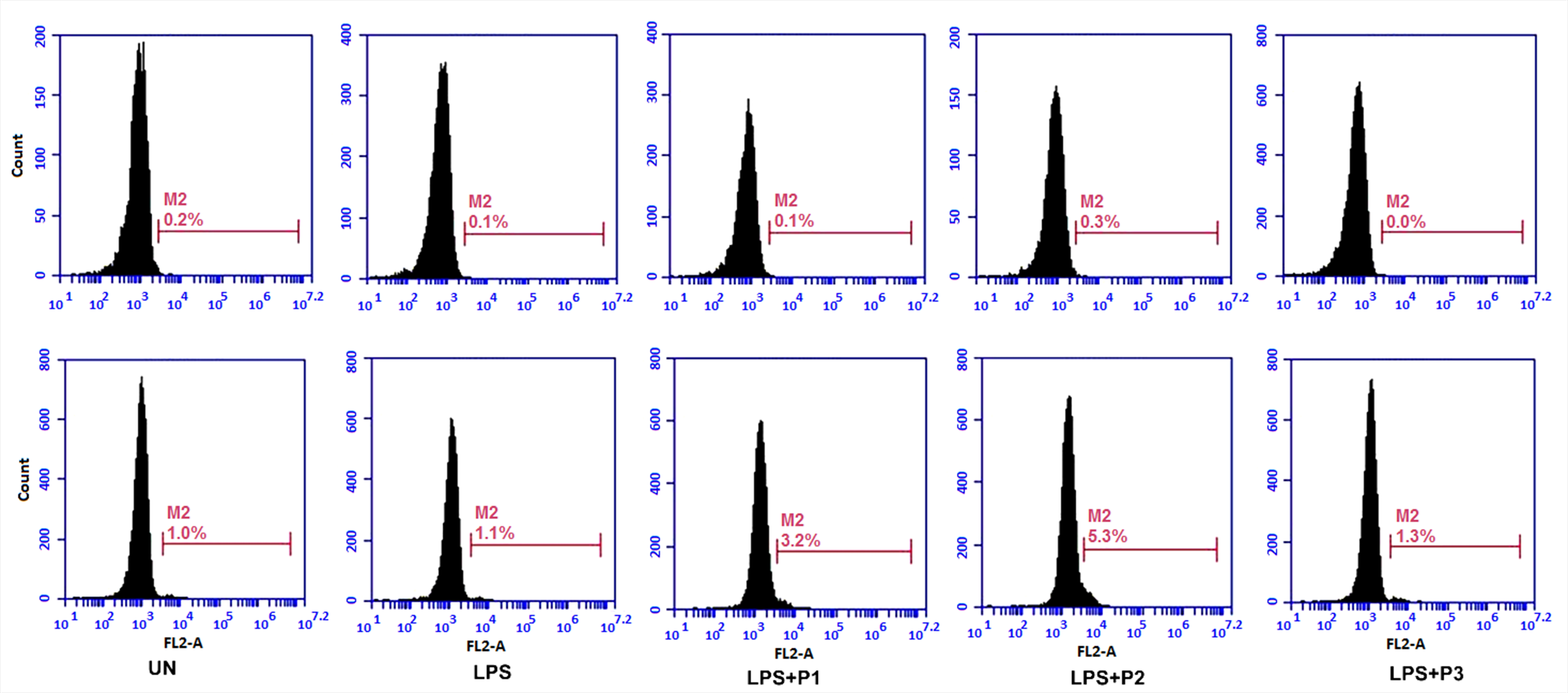

Supplement: S5 Fig — M2 of the X axis is the 7 aad+ cells (dead cells), Y axis represents the cell numbers. (TIF) [file pone.0153939.s005.tif]
