# Supplementary material for: Investigation the Possibility of Using Peptides with a Helical Repeating Pattern of Hydro-Phobic and Hydrophilic Residues to Inhibit IL-10
Source: PLoS One. 2016 Apr 21;11(4):e0153939. doi: 10.1371/journal.pone.0153939 (PMC4839630; doi:10.1371/journal.pone.0153939)
Supplement: S1 Table — (DOCX) [file pone.0153939.s006.docx]

**S1 Table** Hydrogen bonds between IL-10 and IL-10R1 complex [side chain NH (H_N_), hydroxyl hydrogen (H_O_); backbone amide nitrogen (H), backbone carbonyl oxygen (O), and carboxylate oxygen (O_X_), sequences followed the PDB entry 1J7V]

| IL-10 | IL-10R1 | Bond length (Å) |
| --- | --- | --- |
| Arg27: H_N1_ | Ser191: O | 1.91 |
| Arg27: H_N2_ | Ser191: O | 2.01 |
| Lys34: H_N_ | Glu101: O_X_ | 1.81 |
| Gln38: O | Arg76: H_N_ | 1.96 |
| Asp44: O | Gly44: H | 1.95 |
