# Supplementary material for: Investigation the Possibility of Using Peptides with a Helical Repeating Pattern of Hydro-Phobic and Hydrophilic Residues to Inhibit IL-10
Source: PLoS One. 2016 Apr 21;11(4):e0153939. doi: 10.1371/journal.pone.0153939 (PMC4839630; doi:10.1371/journal.pone.0153939)
Supplement: S2 Table — (DOCX) [file pone.0153939.s007.docx]

**Table S2** Hydrophobic interaction regions formed by IL-10 and IL-10R1.

| IL-10 | IL-10R1 |
| --- | --- |
| Pro20-Asp18 | Phe292, Ala189-Ser192 |
| Lys34, Thr35 and Gln38 | Thr93, Asn94 |
| Asp44-Leu46 | Leu41-Glu46, Ser72-Gly74 |
